# Mouse Models for Atherosclerosis Research—Which Is My Line?

**DOI:** 10.3389/fcvm.2019.00046

**Published:** 2019-04-12

**Authors:** Sara Oppi, Thomas F. Lüscher, Sokrates Stein

**Affiliations:** ^1^Center for Molecular Cardiology, University of Zurich, Zurich, Switzerland; ^2^Heart Division, Royal Brompton & Harefield Hospitals and Imperial College, London, United Kingdom

**Keywords:** atherosclerosis, cardiovascular disease (CV disease), mouse models, immunometabolic disease, lipoprotein metabolism, inflammatory signaling, PCSK9 (proprotein convertase subtilisin kexin type 9), Fibrillin 1

## Abstract

Atherosclerosis is one of the primary causes of cardiovascular disease and mortality. This chronic immunometabolic disease evolves during decades in humans and encompasses different organs and immune cell types, as well as local and systemic processes that promote the progression of the disease. The most frequently used animal model to study these atherogenic processes and inter-organ crosstalk in a short time frame are genetically modified mouse models. Some models have been used throughout the last decades, and some others been developed recently. These models have important differences in cholesterol and lipoprotein metabolism, reverse cholesterol transport pathway, obesity and diabetes as well as inflammatory processes. Therefore, the disease develops and progresses differently in the various mouse models. Since atherosclerosis is a multifaceted disease and many processes contribute to its progression, the choice of the right mouse model is important to study specific aspects of the disease. We will describe the different mouse models and provide a roadmap to facilitate current and future atherosclerosis researchers to choose the right model depending on their scientific question.

## Background

**Figure 1:**
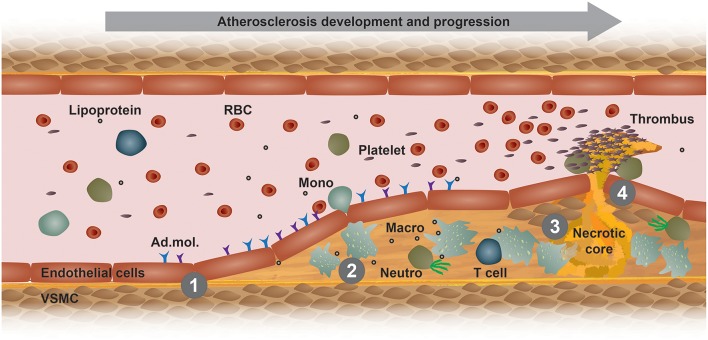
Model of atherogenesis. This scheme illustrates the development of an atherosclerotic plaque from left to right in a longitudinal section of an arterial vessel. (**1**) Upon activation by metabolic or inflammatory triggers, endothelial cells express adhesion molecules (Ad. mol.) that promote the recruitment of immune cells, such as blood monocytes (Mono). These cells then infiltrate the arterial intima, where monocyte differentiate into macrophages (Macro) and interact with other immune cells, such as neutrophils (Neutro) and T cells. (**2**) Increased uptake of modified lipoproteins via scavenger receptors or decreased cholesterol efflux accelerates the accumulation of intracellular free cholesterol and cholesteryl ester-loaded lipid droplets that promote foam cell formation. (**3**) Macrophage foam cells eventually die and fall apart, thereby forming a necrotic core. (**4**) Advanced, vulnerable plaques can rupture and thereby form an arterial thrombus, which can lead to a myocardial infarction or stroke. RBC, red blood cell; VSMC, vascular smooth muscle cell.

Atherosclerosis is a chronic immunometabolic disease and remains asymptomatic until a plaque becomes large enough to obstruct the lumen to cause ischemic pain or ruptures and causes a myocardial infarction, stroke, or peripheral artery disease. At the early stage, the disease is driven by the retention of cholesterol-rich, apolipoprotein B-containing lipoproteins at specific predilection sites such as bifurcations. High level of plasma low-density lipoprotein (LDL)-cholesterol is the most important risk factor promoting the development and progression of atherosclerosis. Lipoproteins that accumulate in the arterial wall undergo various modifications, such as oxidation and carbamylation. These modified lipoproteins and other pro-inflammatory triggers mediate the activation of vascular endothelial cells (Figure 1). In turn, activated endothelial cells express adhesion molecules, which bind to and recruit circulating innate and adaptive immune cells, such as monocytes and T cells (Figure 1). Within the intima, monocytes differentiate into macrophages and ingest modified lipoproteins, becoming cholesterol-laden foam cells (1). Plaque macrophages express different scavenger receptors that recognize and mediate the uptake modified lipoprotein antigens, such as oxidized lipoproteins, hence promoting foam cell formation and a pro-inflammatory polarization (Figure 1) (2). The excessive storage of cholesterol esters leads to a defective esterification pathway, thus resulting in a consistent accumulation of free cholesterol that forms cholesterol crystals that damage the cells and activate apoptotic pathways. Efferocytosis, i.e., the phagocytosis of apoptotic and necrotic cells, gets impaired and promotes a further accumulation of foam cell debris and the release of inflammatory mediators that together potentiate the inflammation of the arterial wall (3). Additionally, foam cells release enzymes that degrade the extracellular matrix, thus increasing plaque vulnerability and the eventual risk of rupture, which would lead to platelet aggregation, blood coagulation and thrombus formation (Figure 1) (1). The development and stability of atherosclerotic plaques is also affected by inflammatory cytokines that are released by different immune cells, such as TNF-α and IFN-γ (4). These released cytokines induce an intra-plaque immune response and promote vascular smooth muscle cells (VSMCs) death, thus destabilizing the matrix of the plaques. Moreover, other cells and organs also contribute to the immunometabolic dysregulation happening during atherosclerosis development. Therefore, it is advisable to compare the different atherosclerosis mouse models and choose one that resembles the aspects of the human pathology as good as possible.

## Animal Models

The pathophysiology of atherosclerosis in humans is a complex process that is triggered by various risk factors, including aging, hyperlipidemia, hypertension and diabetes, which lead to an immunometabolic dysregulation. The study of the immunometabolic processes and molecular mechanisms driving the disease requires animal models that mimic the human pathophysiology. Notably, there is no perfect animal model that recapitulate all the features of the human disease. Several animal models have been studied for atherosclerosis research over the last decades, and all of them show advantages and disadvantages. Different animal models can be chosen depending on the focus of the research. In terms of human physiology similarities and clinical relevance, non-human primates are the best model for atherosclerosis investigation. However, non-human primates are expensive to maintain, they develop the disease over a long time, there is a high risk of infections, and they have high ethical hurdles (5, 6). Alternative animal models should be cheaper, easier to handle and reproduce the human disease as good as possible (7). Moreover, they should be appropriate to perform genetic, pharmacological and/or interventional studies.

## Of Mice and Men

Mouse models meet these criteria at least in part and are thus are the most common animal model used for atherosclerosis studies. Nevertheless, mice also display major genetic and physiological differences compared to humans (8). One of the most evident difference between mice and humans resides in the lipoprotein metabolism. Mice are considered as a high-density lipoprotein (HDL) models since most of the cholesterol is transported in HDL particles, and not in LDL as in humans. Consequently, mice carry most plasma cholesterol in HDL particles and overall have massively lower cholesterol levels, which confers atherosclerosis protection due to an improved reverse cholesterol transport pathway (9). One reason for this difference is the lack of the cholesteryl ester transfer protein (CETP) in mice. In fact, CETP promotes the transfer of cholesterol ester from HDL to very low-density lipoproteins (VLDL) and of triglycerides from VDLD to HDL. Humans display a high expression CETP, which in turn leads to increased VLDL- and LDL-cholesterol levels.

Another important difference between mice and humans resides in the different bile acids composition. Additionally to the classical bile acid species that are synthesized in humans, mice produce α- and β-muricholic acids, which are more hydrophilic and thus reduce the uptake of cholesterol in the intestine (10). Moreover, the different composition of secondary and tertiary bile acids (e.g., tauro vs. glycin conjugation) and increased synthesis of bile acids is another reason for an improved reverse cholesterol transport and fecal cholesterol excretion in mice (11). These differences in lipoprotein metabolism and bile acids composition confer the mice a resistant to develop atherosclerosis.

To bypass these limitations and provide an appropriate model for the pathophysiology of the disease, dietary and genetic manipulations were developed to generate mouse models suitable for atherosclerosis studies. The standard chow diet of mice usually contains a low content of cholesterol (0.02–0.03%) and fats (5–6%). This low lipid content does not suffice to promote the development of atherosclerosis (12). Therefore, scientists use ‘humanized diets’, such as the Western-type, containing around 21% fat and 0.15% cholesterol, or the atherogenic diets, which contain more than 1% cholesterol but the same amount of fat. The administration of a high-fat-diet does not induce atherogenesis in most wild-type mouse strains, but it efficiently induces disease development in atherosclerosis-prone genetic mouse models.

These atherosclerosis-prone genetic mouse models were generated by targeting different genes, and they all trigger atherosclerosis development by altering the lipoprotein profile toward an increased VLDL- and LDL-cholesterol content, thus generating a lipoprotein profile that is comparable to humans (13–17).

**Figure 2:**
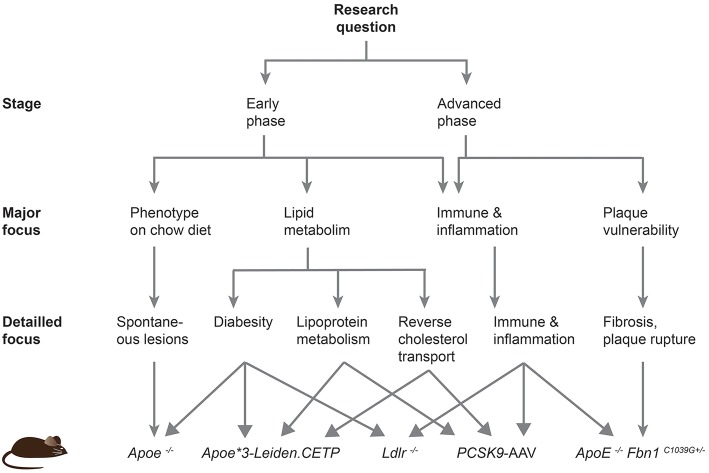
Roadmap to facilitate the choice of an atherosclerotic mouse model. This scheme should help current and future researchers to choose the most appropriate atherosclerotic mouse model based on their specific research question.

Despite the disease development, the predisposed sites for lesion development differ between mice and humans. Plaques in humans preferentially develop in the coronary and carotid arteries, and progress to larger fibrous atheroma. In mice, lesions are mainly localized in the aortic sinus, proximal aorta and aortic arch and brachiocephalic trunk, and do not progress to very advanced stages (18). In fact, even genetically altered mice do not develop plaque rupture or coronary lesions leading to myocardial ischemia or infarction. In this review, we will describe the different transgenic mouse models and provide a roadmap to guide future researchers to choose an adequate model based on the scientific question that needs to be addressed (Figure 2).

## Atherogenic Mouse Strains

Although mice are classified as high-density lipoprotein models, there are important strain-specific differences in lipoprotein metabolism and inflammatory susceptibility. BALB/c, C3H strains have comparable HDL levels, while the C57Bl/6 strain has much lower HDL levels (19). Additionally, C57Bl/6 mice become obese, diabetic and susceptible to lesion development when fed with an atherogenic diet (20). Another important feature that makes the C57BI/6 mice an ideal model for the study of atherosclerosis and metabolic syndrome is the polarization toward the Th1 profile. C57Bl/6 mice contain T cells that mainly produce Th1 cytokines, especially high interferon-γ (IFN-γ), while other strains such as the BALB/c mice release more Th2 cytokines and less IFN-γ (21). The Th1 profile of C57Bl/6 mice makes the model even more pro-atherogenic. Indeed most of the pathogenic T cells in atherosclerosis show a Th1 profile, characterized by the production of high levels of IFN-γ that activate monocytes/macrophages and dendritic cells, increase the expression of matrix metalloproteinases and prevent the collagen disposition from VSMCs, thus destabilizing the fibrous cap and promoting lesion development (22).

## Cholesterol and Lipoprotein Metabolism

Despite of the propensity of the C57Bl/6 strain to develop the disease, different strategies were produced to increase LDL-cholesterol and thereby the atherosclerotic susceptibility of the C57Bl/6 strain, including the disruption of the *low-density lipoprotein receptor* (*Ldlr*-/-), the deletion of the *apolipoprotein E* (*Apoe*-/-), or the ectopic introduction of a mutant *protein convertase subtilisin/kexin type 9* (*Pcsk9*) gene.

APOE is part of the structure of chylomicrons remnants, VLDL and HDL, and it binds to the LDLR, VLDL receptor (VLDLR) and LDLR-related protein (LRP) in the liver to facilitate the clearance of plasma chylomicrons and VLDL remnants. Consequently, the genetic deletion of *Apoe* in mice results in increased levels of plasma cholesterol. *Apoe* knockout mice show a significant increase of total plasma cholesterol compared to wild-type mice under chow diet: 400–600 and 75–110 mg/dl, respectively (23). Of note, humans usually have total plasma cholesterol levels below 200 mg/dl; while levels over 240 mg/dl are considered to be high and are commonly related to familial hypercholesteremia (i.e., mutations in the LDL- or PCSK9 gene). That explains why the deletion of *Apoe* is sufficient to drive a massive hypercholesterolemia under normal diet and to develop spontaneous lesions. Feeding *Apoe*-/- mice with a high cholesterol diet additionally increases plasma cholesterol levels above 1,000 mg/dl, thus driving an extensive and accelerated atherosclerosis development. Lesion distribution in *Apoe*–/– mice are similar to humans, with a predominance in the aortic root, carotid artery, and aortic branches. However, the *Apoe*–/– model shows a different lipoprotein profile from humans since the majority of plasma cholesterol is carried by VLDL and chylomicrons particles, whereas it is mainly transported by LDL in humans. Another limitation of this mouse model is that despite the accelerated atherosclerosis development, the lesions rarely rupture and hence do not lead to thrombosis, whereas vascular occlusion is common in humans (24). In humans, different isoforms of *APOE* are linked to altered lipoprotein profiles and increased cardiovascular mortality (25). Individuals carrying the APOE4 allele have more predisposition to develop cardiovascular disease compared to the APOE2 and APOE3 isoforms. In fact, the APOE4 isoform increases oxidative stress and inflammation, hence promoting disease development (26).

A model closer to the human situation to study altered cholesterol metabolism is the *Ldlr* knockout mouse. The LDLR is a membrane receptor located on the surface of many cell types and its function is to mediate the endocytosis of circulating LDL. The genetic deletion of the *Ldlr* increases cholesterol levels to 200–300 mg/dl on chow diet, and to about 1000 mg/dl on an atherogenic diet (15). The lesions preferentially develop in the proximal aorta at early stages and along the distal aorta at more advanced stages (27). The main aspect that makes *Ldlr* a favorable model to study cholesterol metabolism over the *Apoe* knockouts is its closer resemblance to human hypercholesterolemia since most cholesterol is transported by LDL particles (28). In humans, over 600 mutations of the *LDLR* gene have been reported, several of them causing familial hypercholesterolemia, a frequent genetic disorder associated with high levels of LDL-cholesterol and atherosclerosis development (29).

## Reverse Cholesterol Transport

In the reverse cholesterol transport (RCT) peripheral cholesterol is transferred to HDL particles and then transported to the liver. Within the liver it can then be further metabolized, be converted to bile acids, or be directly excreted via the bile. RCT is therefore regulated at various different sites, including the cholesterol efflux in peripheral tissues and cells, the exchange of lipids between lipoproteins in the blood, and the uptake, metabolization and biliary excretion in the liver (30).

APOE plays a central role in peripheral and hepatic RCT (31). Additionally, macrophages synthesize APOE and promote cholesterol efflux, and interestingly the macrophage-specific deletion of *Apoe* affects peripheral RCT *in vivo* (32–34). Moreover, APOE-positive macrophages promote proper RCT in Apoe knockout mice (34). Therefore, the *Apoe* deletion does affect both peripheral and hepatic RCT. Given the fact that the lipoprotein profile of *Ldlr*–/– mice is closer to humans and considering the presence of APOE in the system, the *Ldlr* knockout is the favored model for RCT and lipoprotein studies.

Another suitable model is the Apoe^*^3-Leiden.CETP mouse line. This model has increased VLDL/LDL-cholesterol because of the expression of a human CETP, reduced peripheral cholesterol efflux, and severe atherosclerosis development (35). Moreover, Apoe^*^3-Leiden.CETP mice fed a high-fat high-cholesterol diet for 6 months mimic changes in lipid profiles observed in humans suffering from the metabolic syndrome, and may therefore be the preferred model to study age-related changes in lipid metabolism and reverse cholesterol transport (36, 37).

## Obesity and Type-2 Diabetes

Obesity, insulin resistance and type-2 diabetes are reaching epidemic proportions world-wide, correlate with atherosclerosis development, and are strong predictors of cardiovascular mortality (38). Currently, there is no ideal mouse model that resembles all features of human diabetes, although several models have been established to study diabetes-accelerated atherosclerosis development, including genetic manipulations and specific diets (39). Diabetogenic diets that are rich in fats and sucrose induce obesity in C57Bl/6 mice, making this strain ideal to study the development of type-2 diabetes and atherosclerosis (40). *Ldlr*–/– mice are more susceptible to develop diabetes compared to the *Apoe*–/– mice and the control wildtype mice under diabetogenic diet feeding. In fact, *Ldlr*–/– mice have an increased body weight caused by an accumulation of subcutaneous adiposity, have high glucose levels and develop an insulin resistance upon diabetogenic diet challenge (41). This diabetic phenotype is not observed in *Apoe*–/– mice, possibly due to an increased rate of hepatic fatty acid oxidation (41). Conversely, on a high fat diet, both *Apoe*–/– and *Ldlr*–/– mice develop features of type 2 diabetes and promote atherosclerosis development (42, 43). Therefore, the choice of the diet is very important in this context.

Further models were generated by crossbreeding genetic models of type-2 diabetes, such as *leptin* (*ob/ob*) and *leptin receptor* (*db/db*) deficient mice, with atherosclerotic mouse models. These genetic models enable the study of the disease development under chow diet (39). Leptins are hormones synthesized by adipocytes, released into the bloodstream, and exerting their main functions by binding to leptin receptors at the hypothalamus to regulate the appetite and thus food intake. Moreover, leptins increase the energy expenditure that translate into an elevation in body temperature and increase in oxygen consumption. Therefore, deficiency of *leptin* or the *leptin receptor* leads an impaired energy expenditure and increased food intake, and thus to obesity (44, 45). Moreover, genetic deficiency of the leptin axis impairs the immune system and increases the susceptibility to infections (46). Thus, *leptin* or the *leptin receptor*-deficient mice display common features of the immuno-metabolic dysfunction that are observed in obesity and diabetes mellitus. Mice that develop both diabetes and atherosclerosis are a good model for the study diabetes-accelerated atherosclerosis development. For example, *Apoe*–/– *db/db* mice develop obesity, hyperinsulinemia, dyslipidemia and have a fast atherosclerosis development at 20 weeks of age compared to the control *Apoe*–/– mice (47). *Ldlr*–/– *ob/ob* mice get obese and show hyperglycemia, hypercholesterolemia and spontaneous lesions development under chow diet (48). In conclusion, multiple mouse models can be used to study diabetes-accelerated atherosclerosis, but the interpretation of the data has to be cautious given the various functions of leptin in many physiological processes. We refer to the review of Wu and Huan for further information on this topic (39).

## Inflammatory Processes

Inflammatory and immune processes play an important function in early steps of plaque formation, but also in advanced stages as reveal by the recent CANTOS trial (49). Lipoprotein oxidation, endothelial cell activation, macrophage activation and impaired efferocytosis, VSMCs proliferation, and platelet aggregation are some of the best characterized processes that contributes to the arterial wall inflammation, lesion expansion, and atherothrombosis. APOE is a multifunctional protein that affects each of these inflammatory processes. Functional APOE protein inhibits lipoproteins oxidation, while *Apoe*–/– mice display increased peroxidation of lipoproteins (50). *Apoe*–/– mice also show elevated levels of endothelial cell adhesion molecules, thus triggering the recruitment of monocytes and thymocytes into the subintimal space (23, 51). Moreover, APOE inhibits VSMC proliferation and migration, and consequently *Apoe*–/– mice display increases proliferation and migration of VSMCs (52). Additionally, APOE inhibits platelet aggregation, hence displaying an additional anti-atherogenic function (53). Efferocytosis, the phagocytic clearance of dead cells and cellular debris, plays important functions in the resolution of inflammation and is mainly mediated by macrophages and other immune phagocytes (54). The APOE protein has been shown to exert important functions in this process both *in vivo* and *in vitro* (55). Indeed, APOE promotes the ingestion of apoptotic cells in macrophages, and thus *Apoe*-deficient mice display impaired efferocytosis and an accumulation of apoptotic cells and cellular fragments in the vessel wall, further promoting lesion development. These anti-inflammatory properties of APOE, combined with its impact on lipoprotein metabolism, explain why *Apoe* knockout mice display a very strong development of atherosclerosis compared to other mouse models. Therefore, other models with a functional APOE protein, such as the *Ldlr*–/–, should be the primary choice to study inflammatory processes in atherosclerosis development.

## Immunometabolic Regulation

Currently, one of the most interesting research questions is to assess the impact of inflammatory and immune processes on lipid and lipoprotein metabolism, and vice versa. The use of the *Apoe*–/– or *Ldlr*–/– models to address this question is limited due to their strong impact on inflammatory processes and/or lipoprotein metabolism. Another model that has been developed recently by two different groups are the *PCSK9*-AAV mouse lines (16, 17). These mice have no genetic modifications and express APOE and LDLR at normal levels. Nevertheless, the introduction of a mouse or human gain-of-function PCSK9 mutant leads to increased total plasma cholesterol (above 1,000 mg/dl) as well as VLDL- and LDL-cholesterol, and the development of atherosclerosis upon Western diet feeding. One further advantage of these lines is that a single adeno-associated virus (AAV) injection is sufficient to generate new mouse models in a much quicker time compared to conventional crossbreeding to *Apoe*–/– or *Ldlr*–/– mice. Another advantage is that immunometabolic processes can be studied without the confounding effects from the lack of APOE or LDLR. Although the use of AAVs seems to be pretty save and without pathogenicity, one might still consider a possible anti-viral host immune response of the organism (56).

## Atherothrombotic Studies

A clear limitation of the above described mouse models is that despite developing atherosclerotic lesions, these lesions rarely progress to advanced stages with atherothrombotic vascular occlusion that are observed in humans. Consequently, no spontaneous plaque rupture is observed in these mouse lines. To overcome this limitation a group of Belgium scientists developed and characterized a very interesting mouse model that displays many features of advanced atherosclerotic plaques (57). The mouse model was created by crossbreeding mice with a mutant fibrillin-1 allele (*Fbn1*^C1039G^) with *Apoe* knockout mice, thus generating *Apoe*^−/−^ *Fbn1*^C1039G+/−^ mice. In these mice, the *Fbn1* mutation leads to the fragmentation of elastic fibers, which in turn promotes arterial stiffening and the development of large vulnerable atherosclerotic plaques that eventually rupture (58). Moreover, these mice display increased inflammation and degradation of the extracellular matrix within plaques, and an increased blood-brain barrier (BBB) permeability that leads to the development of xanthomas in the brain upon prolonged exposure to a Western diet (58, 59). Given its strong phenotype and clinical relevance, this line is currently the most interesting genetic mouse model to study advanced atherosclerosis and atherothrombosis in myocardial infarction or ischemic stroke.

Another mouse model that promotes plaque destabilization and stimulates plaque rupture as well as spontaneous atherothrombosis consists of *Apoe*-deficient mice fed with a high-fat diet for 4 weeks and a subsequent infusion of angiotensin II for 4 weeks. The continuous infusion of angiotensin II accelerates the destabilization and vulnerability of the plaques as well as abdominal aortic aneurysm (60). Angiotensin II increases the blood pressure, recruits monocytes, activates macrophages and increases the oxidative stress (61). Therefore, the *Apoe*-deficient mice fed with high-fat diet and subsequent angiotensin II infusion represents an alternative model to study advanced atherosclerosis.

## Conclusion

The mouse model continues to be the best model organism to decipher the underlying genetic, epigenetic and environmental-induced mechanisms leading to disease development and progression. The *Ldlr* knockout model resembles the human lipoprotein profile pretty well and is therefore a suitable model to study cholesterol and lipoprotein metabolism. Even closer to the human profile are the Apoe^*^3-Leiden.CETP mice, which are certainly the model of choice to study human CETP but also changes in lipid profiles that are also observed in humans suffering from the metabolic syndrome. Obesity and insulin resistance are often associated with type 2 diabetes and increase the risk to develop atherosclerosis. *Ldlr* and *Apoe* knockout mice alone or in combination with a *leptin* or *leptin receptor* deficiency are appropriate to evaluate the metabolic syndrome in diet-induced studies or under normal chow, respectively. Inflammation is another important contributor of the disease development and the *Ldlr*–/– model should preferentially be used over the *Apoe*–/– mice, although other newer models such as the *PCSK9*-AAV approach offer an attractive alternative. Finally, the *Apoe* ^−/−^ *Fbn1*^C1039G+/−^ mouse line is emerging as a new model to study atherothrombosis, myocardial infarction, and ischemic stroke. These mice form large and vulnerable atherosclerotic plaques that eventually rupture. In conclusions, the study of the diverse processes promoting atherosclerosis requires different mouse models, and the provided roadmap should facilitate current and future researchers to choose an adequate mouse model for their studies (Figure 2).

## Perspective

Currently, a very exciting field is to explore the role of long non-coding RNA (lncRNA) in atherosclerosis and cardiovascular diseases (62). For example, the lncRNA *LeXis* regulates hepatic lipid accumulation and plasma cholesterol levels, and thereby decreases atherogenesis in *Ldlr* knockout mice (63), and MeXis, a lncRNA that is highly expressed in mouse macrophages, promotes macrophage cholesterol efflux, HDL-driven reverse cholesterol transport, and thus reduces macrophage foam cell formation and atherosclerosis development (64). Notably, another lncRNA, CHROME, has been identified as an alternatively regulator of the cholesterol efflux in primates and its levels are elevated in plasma and atherosclerotic lesions of individuals with coronary artery disease (65), highlighting the translational value of mouse studies.

Future studies aimed at identifying and describing new triggers and mechanisms regulating atherosclerosis development will develop novel mouse models to address their specific questions. Already now most studies are using tissue-specific overexpression or knockout mouse models, but also starting to address the function of specific mutations and not yet well-described posttranslational protein modifications, such as SUMOylation, in atherogenesis and cardiovascular diseases (66–70). Moreover, the incredibly fast development of the Crispr/Cas9 system led to the development of new mouse and also larger animal models to study atherogenesis and will continue to accelerate basic and translational cardiovascular research (71–76).

## Author Contributions

SO and SS explored the literature. SO, TL, and SS wrote the manuscript.

### Conflict of Interest Statement

The authors declare that the research was conducted in the absence of any commercial or financial relationships that could be construed as a potential conflict of interest.
